# Arrival-Time Detection in Wind-Speed Measurement: Wavelet Transform and Bayesian Information Criteria

**DOI:** 10.3390/s20010269

**Published:** 2020-01-02

**Authors:** Wei Zhang, Zhipeng Li, Xuyang Gao, Yanjun Li, Yibing Shi

**Affiliations:** 1 School of Automation Engineering, University of Electronic Science and Technology of China, Chengdu 611731, China; weizhang@uestc.edu.cn (W.Z.); lizhipeng1202@std.uestc.edu.cn (Z.L.); xuyanggao@std.uestc.edu.cn (X.G.); ybshi@uestc.edu.cn (Y.S.); 2 Center for Information Geoscience, University of Electronic Science and Technology of China, Chengdu 611731, China

**Keywords:** wind speed, arrival time, wavelet transform, bayesian information criteria

## Abstract

The time-difference method is a common one for measuring wind speed ultrasonically, and its core is the precise arrival-time determination of the ultrasonic echo signal. However, because of background noise and different types of ultrasonic sensors, it is difficult to measure the arrival time of the echo signal accurately in practice. In this paper, a method based on the wavelet transform (WT) and Bayesian information criteria (BIC) is proposed for determining the arrival time of the echo signal. First, the time-frequency distribution of the echo signal is obtained by using the determined WT and rough arrival time. After setting up a time window around the rough arrival time point, the BIC function is calculated in the time window, and the arrival time is determined by using the BIC function. The proposed method is tested in a wind tunnel with an ultrasonic anemometer. The experimental results show that, even in the low-signal-to-noise-ratio area, the deviation between mostly measured values and preset standard values is mostly within 5 μs, and the standard deviation of measured wind speed is within 0.2 m/s.

## 1. Introduction

Accurate measurement of wind speed is of considerable significance in many fields [1]. In atmospheric science, accurate measurement of wind speed has a direct impact on accurate weather forecasting [2]. In agriculture, accurate wind-speed measurements contribute to crop cultivation and growth [3]. On an entire high-speed rail line, one anemometer is placed at every mile to measure wind speed and direction for the safety of the trains using that line [4]. In the military, it is necessary to measure wind speed accurately in a complex environment, as it is vitally essential for the precise targeting of weapons [5]. In the manufacturing process of wind tunnels, accurate wind-speed measurement is needed to calibrate wind speed in wind tunnels [6]. At present, prevailing wind-measuring instruments include mechanical anemometers [7], thermal anemometers [8], pitot tube anemometers [9], and ultrasonic wind-measuring instruments [10,11]. A mechanical anemometer has a rotating part that is easily damaged in use. The measuring range of wind speed is limited because of the design principle of the thermal anemometer. When utilizing a pitot-tube anemometer, temperature must be measured, and its magnitude is greatly influenced by environmental factors, while the application conditions are too harsh. In contrast, ultrasonic anemometers are widely utilized because they have no mechanical structure, no start-up wind speed limitation, a wide measurement range, and many other advantages [12].

Recently, there have been many methods utilized in ultrasonic anemometers, such as the phase-difference [13], Doppler [14], and time-difference [15] methods. The Doppler method must measure the current temperature and requires that the air in the measured wind field contains suspended particles, which requires more stringent testing conditions. The phase-difference method is also difficult to implement due to its complex underlying principle. Li et al. proposed an array signal processing method to design an ultrasonic anemometer [16], but only simulation experiments were carried out. Its practicability must still be studied further.

The time-difference method is a mainstream approach adopted in ultrasonic anemometers that includes a direct time-difference method and a cross-correlation time-difference method [17]. As mentioned above, the key to the time-difference method is determining the arrival time of the ultrasonic echo signal accurately. The commonly used methods for measuring the arrival time of an echo signal include the peak-value, information criteria [18], and Teager-Kaiser energy operator (TKEO) [19] methods. The peak-value method is widely applied in anemometers. It utilizes the time corresponding to the sampling point with the largest amplitude in the echo as the arrival time of the echo signal, but this method is so subjective and open to being affected by noise. The information criteria function method, which was utilized to determine the location of acoustic emissions (AEs) from concrete by Kurz et al. [20], finds the envelope of the signal first, and then determines the arrival time of the echo by calculating the Akaike information criterion (AIC) function of the envelope of the signal. Furthermore, Liu et al. utilize the Hilbert-Huang Transform (HHT) and AIC function to determine the arrival time of the impact signal [21]. They divide the signal into two local stationary auto-regressive (AR) processes that correspond to the noise part before the signal arrives and the signal itself. After determining the order of the AR model, the time corresponding to the minimum value of the AIC function is the arrival time of the echo signal. However, in the case of long-time series, the method needs a complex multi-independent variables fitting model to increase the fitting accuracy, which aggravates the computational complexity. The TKEO method, which is adopted to determine the arrival time of P and S waves in earthquakes by Ismail et al. [22], is based on a hybrid usage of empirical mode decomposition and TKEO algorithms.

For feature recognition of non-stationary signals, many signal processing methods have been advanced. The wavelet transform (WT) is a superior algorithm among them [23,24,25] which has multi-resolution characteristics and enables a target signal to be observed from coarse to fine. In this paper, according to the non-stationarity of an ultrasonic echo signal, we propose a method for determining the arrival time of ultrasonic echo signals based on the WT and Bayesian information criteria (BIC). First, the WT is utilized for the time-frequency analysis of noisy signals to obtain the time-frequency distribution. Then, the BIC function is calculated, and its sampling point with the minimum value of the function corresponds to the arrival time of the echo signal. To verify the accuracy and stability of this method, a three-dimensional ultrasonic anemometer is designed to compare the method with three other methods in a wind tunnel. The experimental results show the advantages of the proposed method.

## 2. Method

### 2.1. Time-Frequency Location Analysis

**Figure 1:**
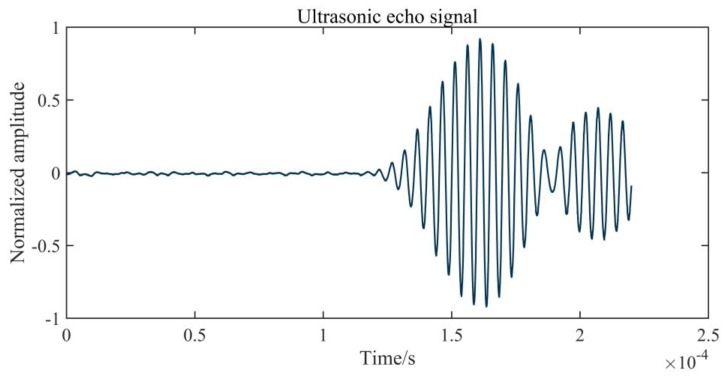
A typical ultrasonic echo signal.

The ultrasonic echo signal is considered to be non-stationary. Its characteristic is that it has two parts: a non-signal part containing only noise before the signal arriving, and an effective part after the signal arrives. Because the acoustic signal excited by the ultrasonic transducer has a single frequency, the echo signal is also of single frequency. A typical echo signal is depicted in Figure 1. Noise, which is considered to be random and uncorrelated to the echo signal, is sampled by sensors before the arrival of the echo signal. The sensor is based on the mechanical principle, and it produces a tail vibration that makes it impossible for the echo to correspond with the transmitted acoustic wave after receiving the ultrasonic signal, as shown in Figure 1. Therefore, the best way to recognize the ultrasonic echo signal is to determine the arrival time of the first wave.

**Figure 2:**
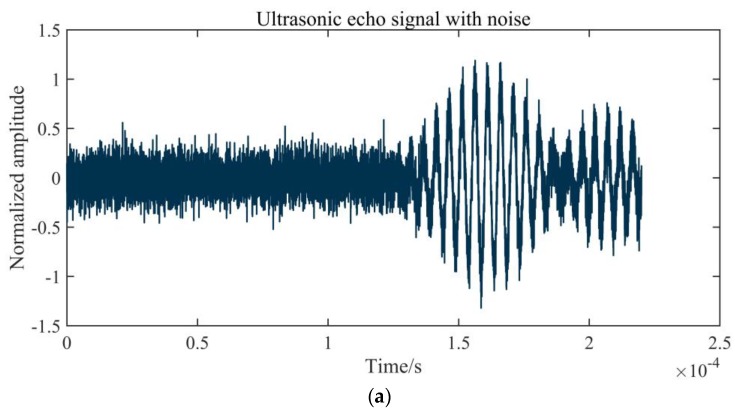

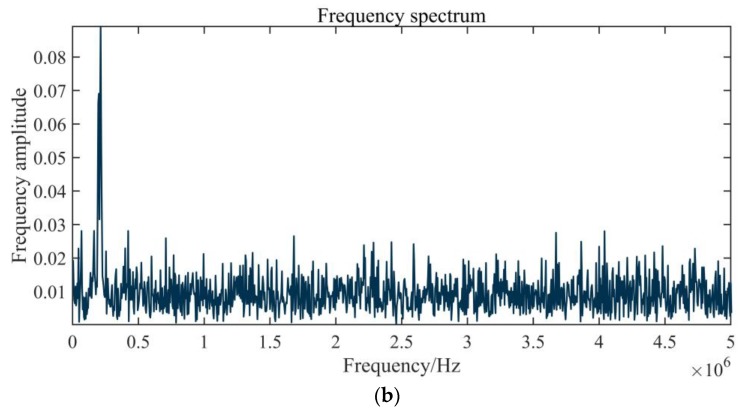
Influence of noise on an echo signal: (**a**) an echo signal with noise; (**b**) the signal frequency spectrum.

However, the frequency spectra of the noise and echo signal overlap significantly in frequency range. In a complex environment, noise sources, such as strong electromagnetic fields and high temperature, inevitably interfere with the instrument [26]. Figure 2 shows a group of typical echo signals in a wind tunnel with electromagnetic interference. In Figure 2a, the amplitude of background noise has exceeded the amplitude of the first wave, and because of the existence of electromagnetic interference, the echo waveform exhibits some distortion. The spectrum of background noise is uniformly distributed in the entire frequency domain, as is shown in Figure 2b, which demonstrates that the arrival time of the first wave cannot be clearly identified, and the filter cannot effectively eliminate the noise.

To accurately determine the arrival time of the first wave in noise, we must utilize parameters with distinct characteristics when the first wave arrives. As mentioned above, it can be seen that the frequency of the signal changes significantly when the echo arrives, so the joint time-frequency analysis can be utilized to estimate the arrival time of the first wave. When the echo signal arrives, the frequency of the signal increases rapidly with time, which means that when the first wave arrives, the joint time-frequency analysis requires good time resolution to provide accurate time-frequency positioning. This can be achieved by pre-processing the signal using the WT. That involves using a signal scalogram to carry out time-frequency analysis and obtain the distribution relationship between time, frequency, and energy.

The WT, proposed by Morlet [26], can provide a time-frequency window that changes with frequency. The definition of a continuous WT is expressed as

(1) $$Wx\left(a,b\right)=\frac{1}{\sqrt{a}}\int_{-\infty }^{+\infty }x\left(t\right)\psi^{*}\left(\frac{t-b}{a}\right)dt,$$

where a is a scale factor associated with frequency and b is a displacement factor that describes wavelet movement in the time domain. A mother wavelet generates a function family $\psi_{a,b}\left(t\right)=\frac{1}{\sqrt{a}}\psi \left(\frac{t-b}{a}\right)$ by changing a and b. x(t) is an echo signal that changes persistently and ψ(t) is the mother wavelet that must satisfy the following conditions:

(2) $$\int_{-\infty }^{+\infty }\psi \left(t\right)dt=0.$$

In addition, a Fourier transform (FT) Ψ(ω) of wavelet function ψ(t) must satisfy the following conditions:

(3) $$C_{\psi }=\int_{-\infty }^{+\infty }\frac{{\left|\Psi \left(\omega \right)\right|}^{2}}{\left|\omega \right|}d\omega <\infty$$

**Figure 3:**
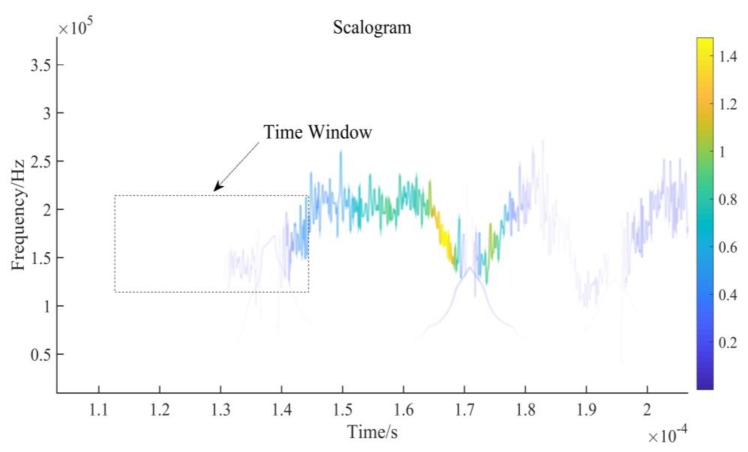
Time-frequency distribution based on Morlet WT (MWT).

The selection of the wavelet function is essential in the WT, which is related to whether good resolution can be obtained in the time-frequency domain. The frequency of the echo signal received by the sensor is a single one, which means a high temporal resolution is required for time localization of this single frequency. The Morlet WT (MWT), which utilizes the Morlet wavelet function, is an ideal option [27], and its analytic formula is

(4) $$\psi \left(t\right)=e^{j\omega_{0}t}e^{-\frac{t^{2}}{2}},\;\;\omega_{0}\geq 5,$$

where $\omega_{0}$ is the wavelet central frequency and j an imaginary number unit. Therefore, Equation (1) can be rewritten as

(5) $${\left|Wx(a,b)\right|}^{2}\;=\;{\left|\frac{1}{\sqrt{a}}\int_{-\infty }^{+\infty }x(t)e^{-jw_{0}\frac{t-b}{a}}e^{-\frac{{\left(t-b\right)}^{2}}{2a^{2}}}dt\right|}^{2},$$

where ${\left|Wx\left(a,b\right)\right|}^{2}$ is a scalogram of the echo signal that denotes the energy distribution by displacement factor b and scale factor a. The scale factor a is related to the frequency f as expressed by

(6) $$f=\frac{\omega_{0}f_{S}}{2\pi a},$$

where $f_{S}$ is the sampling frequency. Thus, Equation (5) can be rewritten as

(7) $${\left|Wx\left(f,b\right)\right|}^{2}={\left|\sqrt{\frac{2\pi f}{\omega_{0}f_{S}}}\int_{-\infty }^{+\infty }x\left(t\right)e^{-j\frac{2\pi f\left(t-b\right)}{f_{S}}}e^{-\frac{2{\left[\pi f\left(t-b\right)\right]}^{2}}{{\left(\omega_{0}f_{S}\right)}^{2}}}dt\right|}^{2},$$

according to which the time-frequency relationship of the typical echo signal can be obtained and the time-frequency distribution of the echo signal drawn as in Figure 3.

Figure 3 shows the MWT of Figure 2a. In Figure 3, the time window clearly shows that the energy changes significantly with signal frequency when the echo signal arrives. Tests on numerous amounts of noise show the same regularity. Based on this characteristic, sampling points that change with abrupt energy can be found on the echo signal, which can be utilized as a reference to find the precise arrival time of the echo signal.

### 2.2. Accurate Arrival-Time Determination of Echo Signal Based on BIC

The sampling points obtained by the WT in the time window contain the precise arrival time of the echo signal, and the real arrival time needs further precise positioning. As mentioned in the Introduction, the BIC function is applied to the pre-processed result using a certain method that is defined as

(8) BIC=−2ln(L)+klnn,

where L is a likelihood function, k the order of the model, and n the number of sampling points [28]. To distinguish noise from the echo signal, one ideal method combines the AR model and BIC to obtain accurate sampling points of first wave [28,29,30]. The *m*th-order AR model is expressed as [30]

(9) $$u_{t}=\sum_{i=1}^{m}a_{i}u_{t-i}+\varepsilon_{t}\;,$$

where: $\varepsilon_{t}$ is Gaussian white noise with zero mean, and its variance is $\sigma^{2}$; $a_{i}$ is the AR coefficient; and $u_{t}$ denotes the discrete ultrasonic echo signal acquired by an analog-to-digital converter. $\varepsilon_{t}$ also obeys the normal distribution, which means that the probability density function of it is [31]

(10) $$\rho \left(\varepsilon_{t}\right)=\frac{1}{\sqrt{2\pi \sigma^{2}}}e^{-\frac{\varepsilon_{t}^{2}}{2\sigma^{2}}}.$$

The rough sampling point P, which corresponds to the arrival time of the first wave and is obtained by the MWT, is defined as $S_{2}$. Before and after the point P, part of the echo signal is cut out as the time window that contains sampling points, and its length is N. Thus, the starting point of the acquisition window, which is defined as $S_{1}$, is P−0.5N, and the ending point of the window, which is defined as $S_{3}$, is P+0.5N. M(1) and M(2) denote the orders of the AR model before and after the P point, respectively.

Therefore, according to Equations (9) and (10) and the definition of the likelihood function [32,33], the likelihood function of all sampling points in the entire time window is

(11) $$L\left(a^{i},\;\sigma_{i}^{2}\right)=\prod_{i=1}^{2}{\left(\frac{1}{2\pi \sigma_{i}^{2}}\right)}^{\frac{\Delta N_{i}}{2}}exp\left(-\frac{1}{2\sigma_{i}^{2}}\sum_{t=h_{i}}^{s_{i}}{\left(x_{t}-\sum_{m=1}^{M\left(i\right)}a_{m}^{i}x_{t-m}\right)}^{2}\right),$$

where $\Delta N_{i}$ is defined as $S_{i}-S_{i-1}$, $h_{1}$ is P−0.5N, $h_{2}$ is P+1, $x_{t}$ is the discrete ultrasonic echo signal, and $\sigma_{i}$ is the variance of the noise corresponding to the AR model of order M(i). The maximum value of Equation (11) is the maximum likelihood estimation of the echo signal [31,33], which is

(12) $$L\left(\sigma_{i}^{2}\right)=-\frac{N}{2}\left(1+\ln2\pi \right)-\frac{1}{2}\sum_{i=1}^{2}\Delta N_{i}\sigma_{i}^{2},$$

where $\sigma_{i}^{2}$ equals $\frac{1}{\Delta N_{i}}\sum_{t=S_{i}}^{K}{\left(x_{t}-\sum_{m=1}^{M\left(i\right)}a_{m}^{\left(i\right)}x_{t-m}\right)}^{2}$ when the derivative of Equation (11) is 0 [31].

**Figure 4:**
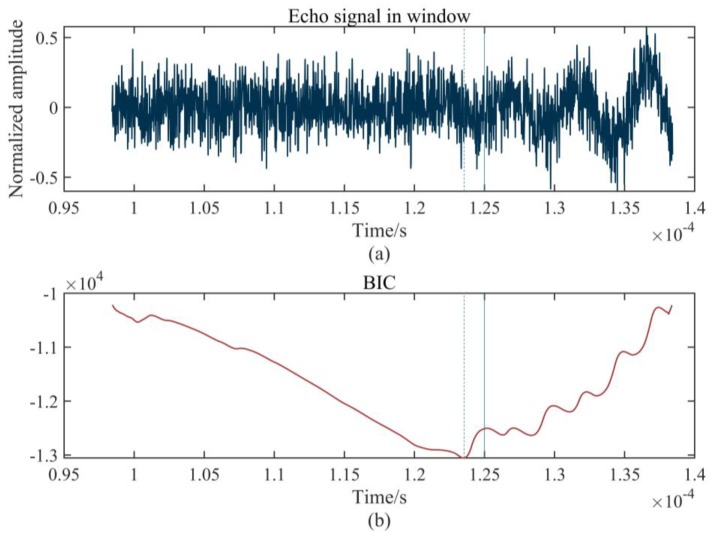
Time-frequency distribution based on MWT: (**a**) time window; (**b**) BIC function.

Thus, according to Equations (8) and (12), the AR-BIC picker of the echo signal can be obtained as

(13) $$\begin{array}{c} BIC\left(K,M\right)=N\left(1+\ln2\pi \right)+\Delta N_{1}\ln\left[\frac{1}{\Delta N_{1}}\sum_{t=S_{1}}^{K}{\left(x_{t}-\sum_{m=1}^{M\left(1\right)}a_{m}^{\left(1\right)}x_{t-m}\right)}^{2}\right]\\ +\;\Delta N_{2}\ln\left[\frac{1}{\Delta N_{2}}\sum_{t=k+1}^{S_{3}}{\left(x_{t}-\sum_{m=1}^{M\left(2\right)}a_{m}^{\left(2\right)}x_{t-m}\right)}^{2}\right]+\left(\sum_{i=1}^{2}M\left(i\right)\right)\ln N\\ =N\left(1+\ln2\pi \right)+\Delta N_{1}\ln\sigma_{1}^{2}+\Delta N_{2}\ln\sigma_{2}^{2}+\left(\sum_{i=1}^{2}M\left(i\right)\right)\ln N, \end{array}$$

where K is the range through all sampling signal points in the time window. According to references [21,28,31,34], the echo signal received by sensors can be considered as the pure echo signal with added Gaussian white noise, and the noise is uncorrelated with the echo signal. Thus, the variance of the noise, $\sigma_{i}$, can be regarded as the variance of the echo signal received by sensors, and Equation (13) can be rewritten as

(14) $$\begin{array}{c} BIC\left(K,M\right)=N\left(1+\ln2\pi \right)+\Delta N_{1}\ln\left[var\left(x\left(S_{1},K\right)\right)\right]\\ +\Delta N_{2}\ln\left[var\left(x\left(K+1,S_{2}\right)\right)\right]+\left(\sum_{i=1}^{2}M\left(i\right)\right)\ln N\;, \end{array}$$

where $var\left(x\left(S_{1},K\right)\right)$ denotes the variance of the corresponding interval from $S_{1}$ to K in the time series x(t). When Equation (14) takes minimum values, it determines the arrival time of the echo signal accurately, which is shown in Figure 4.

**Figure 5:**
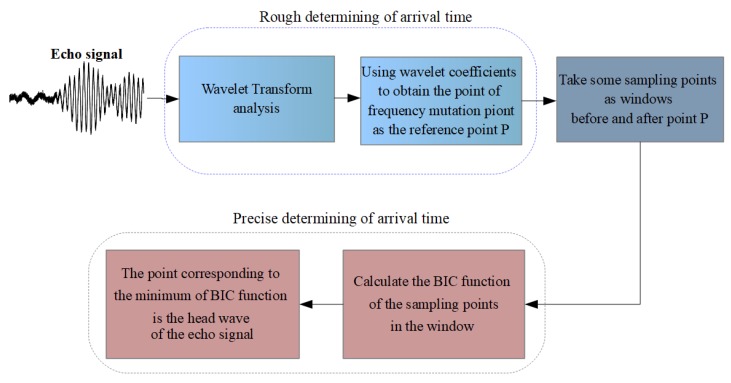
Block diagram of the proposed method.

Figure 4a shows the time window containing the precise arrival time of the echo signal and Figure 4b is the corresponding BIC function. The arrival time of the echo signal indicated by the dotted line is 0.124 ms, which is measured by the proposed method, while the actual determined time of the echo signal without noise is 0.125 ms, which is indicated by a vertical straight line. It can be seen from Figure 4a that the noise has a major influence on the echo signal, so that the characteristics of the signal cannot be identified directly. However, the BIC function can effectively identify the original characteristics of the signal from the noise and accurately find the arrival time of the echo signal, as shown in Figure 4b. The result indicates the satisfactory precision of our method. Figure 5 summarizes the proposed method.

## 3. Experiments and Results

### 3.1. Experimental Platform

**Figure 6:**
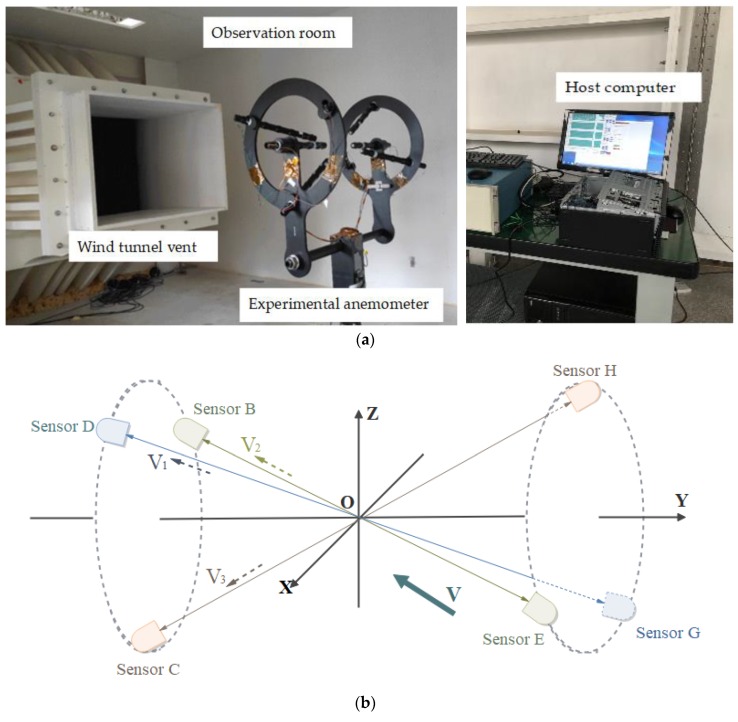

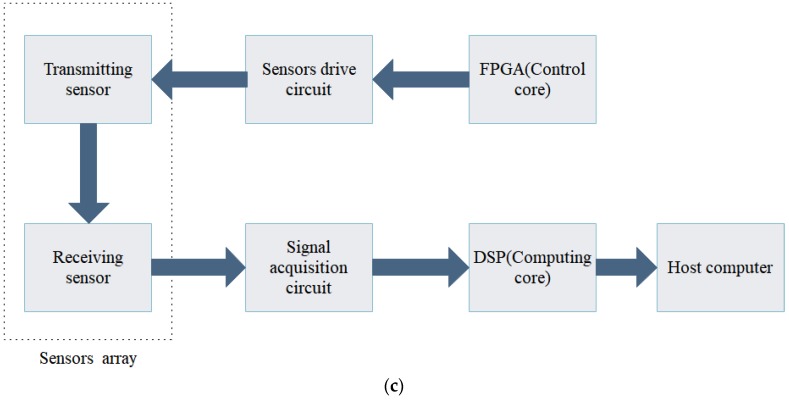
Hardware comprising experimental platform: (**a**) anemometer in wind tunnel; (**b**) sensor positions; (**c**) overall structure of experimental anemometer.

To test the proposed method, a three-dimensional ultrasonic anemometer was designed, consisting of a three-dimensional nonorthogonal ultrasonic sensor array, echo signal acquisition circuit, control and computing core, ultrasonic sensor drive circuit, and host computer, which is shown in Figure 6c. The ultrasonic sensor array is composed of three pairs of ultrasonic sensors that emit the ultrasound in sequence and receive it from the corresponding sensors. All sensors are Airmar AT200 type sensors with a working frequency of 200 kHz. As shown in Figure 6a, the experiment was carried out in the Low-Speed Wind Tunnel at the China Aerodynamics Research and Development Center.

### 3.2. Performance under Different Signal-To-Noise Ratios

**Figure 7:**
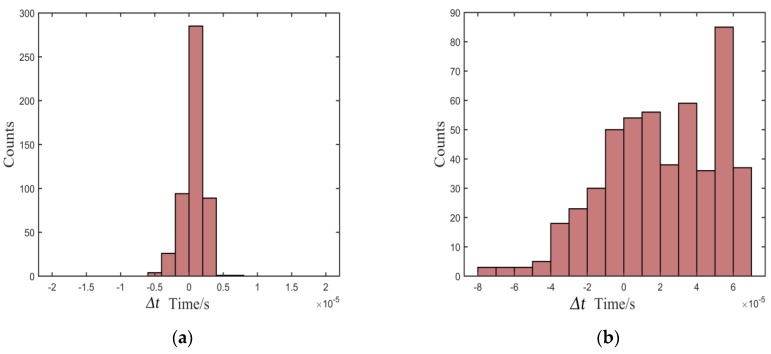

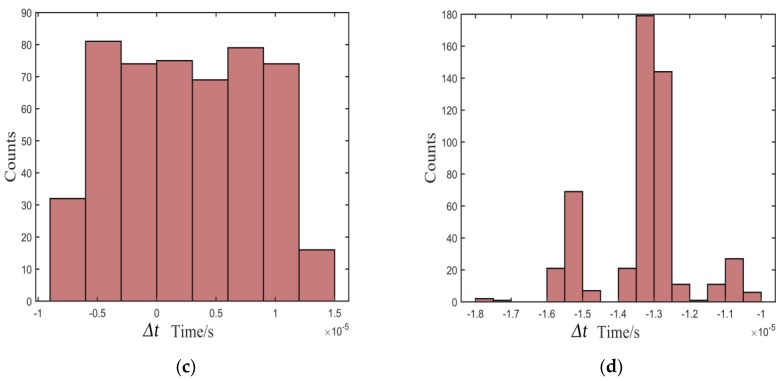
Distribution of deviation in the environment of 10 dB: (**a**) proposed, (**b**) Kurz, (**c**) TKEO, and (**d**) high-order statistics methods.

**Figure 8:**
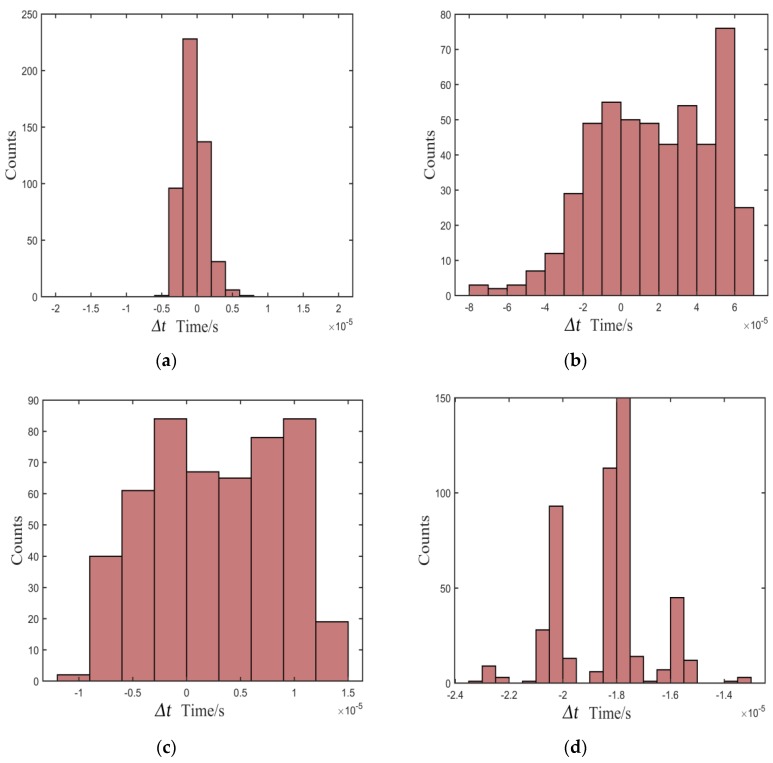
Distribution of deviation in the environment of 5 dB: (**a**) proposed, (**b**) Kurz, (**c**) TKEO, and (**d**) high-order statistics methods.

**Figure 9:**
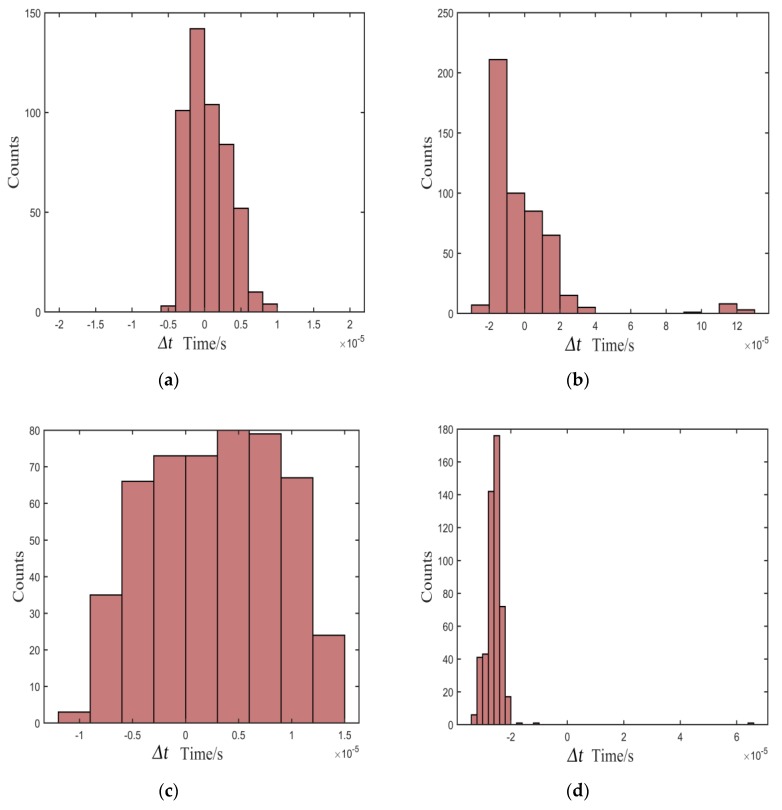
Distribution of deviation in the environment of 0 dB: (**a**) proposed, (**b**) Kurz, (**c**) TKEO, and (**d**) high-order statistics methods.

To test the stability of the proposed method, we designed several actual tests. We fixed the distance of one pair of sensors and measured the standard arrival time of the echo signal in the noiseless environment. Then, 500 tests were carried out under different signal-to-noise ratios (SNRs) to obtain the measured arrival times using the proposed method. For comparison, the Kurz, TKEO, and high-order statistics methods were carried out in the same test environment. Figure 7, Figure 8 and Figure 9 show the distribution of deviation between measured time *T_measured_* and standard time *T_standard_* denoted by Δ*t* = *T_standard_* − *T_measured_*, which is obtained from the proposed, the Kurz, and the TKEO methods, as well as the high-order statistics method under experimental conditions of 10, 5, and 0 dB, respectively. The abscissa denotes *t* Δ*t*, and the ordinate denotes the number of each Δ*t* in Figure 7, Figure 8 and Figure 9.

As shown in Figure 7, Figure 8 and Figure 9, most of the deviations distribute close to zero and are less than ±5 μs using the proposed method, while the distribution is quite intensive. In addition, the distribution shows great stability as the SNR decreases. In contrast, the distributions of Δ*t* are out of the range of ±15 μs using the Kurz, TKEO, and high-order statistics methods, which means that the measured results acquired from the three methods are more dispersive than those obtained utilizing the proposed method, and are more likely to have a relatively larger error in determining the arrival time of the echo signal.

### 3.3. Stability and Accuracy Tests of Wind Speed in Wind Tunnel

**Figure 10:**
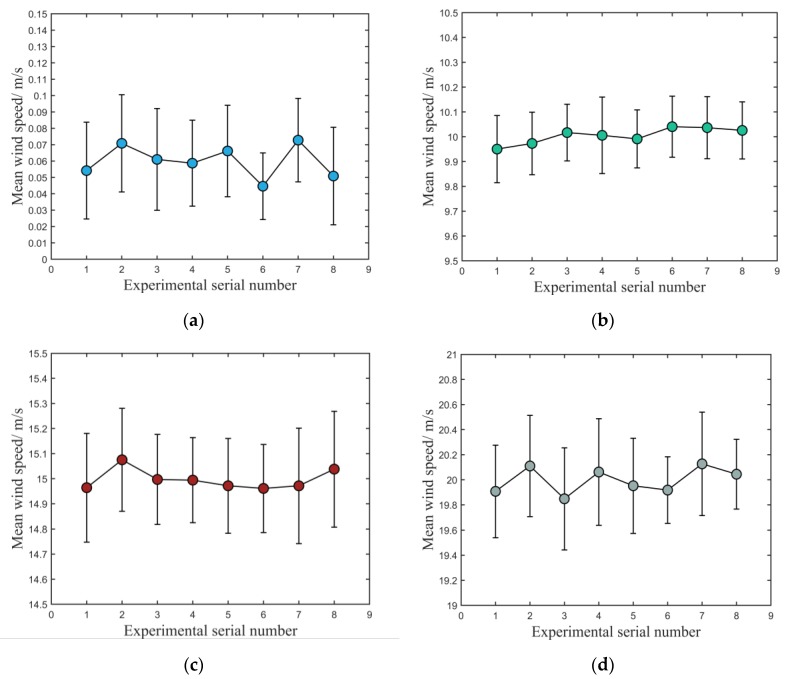
Tests with different wind speeds: (**a**) 0, (**b**) 10, (**c**) 15, and (**d**) 20 m/s.

To test the stability and accuracy of the proposed method in actual wind-speed measurements, three pre-set wind speeds were measured with the above-described ultrasonic anemometer in a wind tunnel. The wind speeds were preset to four separate levels in the wind tunnel: 0, 10, 15, and 20 m/s. Eight tests were carried out at each wind speed, and 150 wind speed data continuously measured in each test. The ambient temperature and relative humidity in the wind tunnel were 16.3 °C and 58%, respectively. The average and standard deviations of a series of wind speeds obtained from each continuous test were calculated, as shown in Figure 10 and Table 1.

Figure 10a shows the experiment without opening the wind tunnel, in which the theoretical wind speed was 0 m/s. According to reference [35], when the wind tunnel is not opened, the measured wind speed within 0.1 m/s can be regarded as being 0 m/s. The results shown in Figure 10a indicate that this method can accurately measure the arrival time of the echo signal and has a strong anti-noise ability for background noise. Figure 10b–d show the results of testing the actual wind speed in the wind tunnel when the pre-set wind speeds were 10, 15, and 20 m/s, respectively. In each wind-speed test, seen from the viewpoints of the average and standard deviation of those experiments, the deviation between the mean value and the wind speed produced by the wind tunnel is within 0.16 m/s, and the standard deviation is within 0.2 m/s, which means that the proposed method has great accuracy and stability.

### 3.4. Discussion of Results of Wind-Speed Tests

It can be found from Figure 10 that the standard deviation of test results increases with increasing wind speed in the wind tunnel. After carefully checking the anemometer, it is found that the bracket carrying the ultrasonic sensors has installation tolerance. With an increasing wind speed, the bracket oscillates to a certain extent, which leads to waveform distortion when the sensor receives the ultrasonic echo signal.

Since the anemometer is a prototype instrument utilized to verify the method, we plan to redesign a new bracket with ultrasonic sensors installed to ensure that this phenomenon will not re-occur.

## 4. Conclusions

In this paper, a new method is proposed to determine the precise arrival time of an ultrasonic echo signal. The method is based on the WT and BIC. The time-frequency distribution of echo signals with strong background noise is obtained by the WT, and the time corresponding to the beginning of frequency variation is determined as the rough position of arrival time. Then, based on the rough position, a segment containing sampled points is cut out before and after the rough point as the time window. Finally, the BIC function of the echo signal in the time window is calculated, and the time corresponding to the minimum BIC-function value is determined as the precise arrival time of the ultrasonic echo signal.

To verify the proposed method, we designed an ultrasonic anemometer and tested it in a wind tunnel. To demonstrate the stability and anti-noise ability of the proposed method, the Kurz, TKEO, and high-order statistics methods were compared. After 500 experiments, the distribution of the deviation between the statistical measured value and pre-supposed standard value show that our method is much more accurate in determining the arrival time. Moreover, the actual different wind speeds in the wind tunnel were measured by the proposed method, and the results also verify that the proposed method performs satisfactorily in terms of stability and accuracy.

## Figures and Tables

**Table 1:** Mean measured values and standard deviations at different wind speeds.

Wind Speed in Wind Tunnel (m/s)	Mean Measured Value (m/s)	Standard Deviation (m/s)
0	0.07	0.03
10	10.15	0.09
15	15.06	0.16
20	20.16	0.19

## References

[1] Deaves D.M., Lines I.G. (1997). On the fitting of low mean windspeed data to the Weibull distribution. J. Wind Eng. Ind. Aerodyn..

[2] Broster J.C., Robertson S.M., Dehaan R.L., King B.J., Friend M.A. (2012). Evaluating seasonal risk and the potential for windspeed reductions to reduce chill index at six locations using GrassGro. Anim. Prod. Sci..

[3] Chiew F.H.S., Kamaladasa N.N., Malano H.M., Mcmahon T.A. (1995). Penman-Monteith, FAO-24 reference crop evapotranspiration and class-A pan data in Australia. Agric. Water Manag..

[4] Tabler R.D. (1979). Visibility in Blowing Snow and Applications in Traffic Operations. Transp. Res. Board Spec. Rep..

[5] Tarade R.S., Katti P.K. A comparative analysis for wind speed prediction. Proceedings of the 2011 International Conference on Energy, Automation and Signal.

[6] Morel T. (1975). Comprehensive Design of Axisymmetric Wind Tunnel Contractions. J. Fluids Eng..

[7] Hyson P. (1972). Cup Anemometer Response to Fluctuating Wind Speeds. J. Appl. Meteorol..

[8] Zhu Y., Chen B., Qin M., Huang Q.A. (2017). 2-D Micromachined Thermal Wind Sensors—A Review. IEEE Internet Things J..

[9] Shi W., Wei S. (1992). Comparison between Pitot Tube and Hot-wire Anemometer in Slow Periodic Flows. J. Exp. Mech..

[10] Bucci G., Ciancetta F., Fiorucci E., Gallo D., Luiso M. A low-cost ultrasonic wind speed and direction measurement system. Proceedings of the Instrumentation & Measurement Technology Conference.

[11] Lopes G.M.G., Junior D.P.D.S., Franca J.A.D., Franca M.B.D.M., Ribeiro L.D.S., Moreira M., Elias P. (2017). Development of 3-D Ultrasonic Anemometer with Nonorthogonal Geometry for the Determination of High-Intensity Winds. IEEE Trans. Instrum. Meas..

[12] Wang H., Tao G., Shang X.F. (2016). Understanding acoustic methods for cement bond logging. J. Acoust. Soc. Am..

[13] Villanueva J.M.M., Catunda S.Y.C., Tanscheit R. (2009). Maximum-Likelihood Data Fusion of Phase-Difference and Threshold-Detection Techniques for Wind-Speed Measurement. IEEE Trans. Instrum. Meas..

[14] Tamura Y., Suda K., Sasaki A., Iwatani Y., Fujii K., Ishibashi R., Hibi K. (2001). Simultaneous measurements of wind speed profiles at two sites using Doppler sodars. J. Wind Eng. Ind. Aerodyn..

[15] Kang J.W., Chu Y.B., Feng H.B. (2012). Study on Ultrasonic Anemometer Measurement System Based on ARM. Instrum. Tech. Sens..

[16] Li X., Sun H., Gao W., Shi Y., Liu G., Wu Y. (2016). Wind speed and direction measurement based on arc ultrasonic sensor array signal processing algorithm. ISA Trans..

[17] Li Y., Wang B., Wu Y. Time-difference Ultrasonic Wind Detection Methods Based on Cross-correlation Theory. Proceedings of the International Conference on Electronic Measurement & Instruments.

[18] Konishi S., Kitagawa G. (2008). Bayesian Information Criteria. Information Criteria and Statistical Modeling.

[19] Khaldi K., Boudraa A.O., Komaty A. (2014). Speech enhancement using empirical mode decomposition and the Teager-Kaiser energy operator. J. Acoust. Soc. Am..

[20] Kurz J.H., Grosse C.U., Reinhardt H.W. (2005). Strategies for reliable automatic onset time picking of acoustic emissions and of ultrasound signals in concrete. Ultrasonics.

[21] Liu M., Yang J., Cao Y., Fu W., Cao Y. (2017). A new method for arrival time determination of impact signal based on HHT and AIC. Mech. Syst. Signal Process..

[22] Kirbas I., Peker M. (2016). Signal detection based on empirical mode decomposition and Teager–Kaiser energy operator and its application to P and S wave arrival time detection in seismic signal analysis. Neural Comput. Appl..

[23] Li H., Manjunath B.S., Mitra S.K. (1995). Multisensor Image Fusion Using the Wavelet Transform. Graph. Models Image Process..

[24] Sinha S., Routh P.S., Anno P.D., Castagna J.P. (2008). Spectral decomposition of seismic data with continuous-wavelet transform. Geophysics.

[25] Zhang B.L., Lv J., Li J.R. (2018). A Compound Algorithm for Parameter Estimation of Frequency Hopping Signal Based on STFT and Morlet Wavelet Transform. International Conference on Intelligent Computing.

[26] Morlet J. (1983). Sampling Theory and Wave Propagation. Issues in Acoustic Signal—Image Processing and Recognition.

[27] Kronlandmartinet R., Morlet J., Grossmann A. (1987). Analysis of sound patterns through wavelet transforms. Int. J. Pattern Recognit. Artif. Intell..

[28] Burnham K.P., Anderson D.R. (2004). Multimodel Inference Understanding AIC and BIC in Model Selection. Sociol. Methods Res..

[29] Mousavi S.R., Niknazar M., Vahdat B.V. Epileptic Seizure Detection using AR Model on EEG Signals. Proceedings of the International Biomedical Engineering Conference.

[30] Leonard M., Kennett B.L.N. (1999). Multi-component autoregressive techniques for the analysis of seismograms. Phys. Earth Planet. Inter..

[31] Sullivan E.J. (2011). Statistical Signal Processing.

[32] He J., Jones J.W., Graham W.D., Dukes M.D. (2010). Influence of likelihood function choice for estimating crop model parameters using the generalized likelihood uncertainty estimation method. Agric. Syst..

[33] Juang B.H., Rahim M.G. (1996). Signal bias removal by maximum likelihood estimation for robust telephone speech recognition. IEEE Trans. Speech Audio Process..

[34] Posada D., Buckley T.R. (2004). Model selection and model averaging in phylogenetics: Advantages of akaike information criterion and bayesian approaches over likelihood ratio tests. Syst. Biol..

[35] Knight I.K. (2001). The Design and Construction of a Vertical Wind Tunnel for the Study of Untethered Firebrands in Flight. Fire Technol..

